# Delayed Blastocyst Formation Reduces the Quality and Hatching Ability of Porcine Parthenogenetic Blastocysts by Increasing DNA Damage, Decreasing Cell Proliferation, and Altering Transcription Factor Expression Patterns

**DOI:** 10.3390/jdb12040026

**Published:** 2024-10-01

**Authors:** Ling Sun, Yan Wang, Mo Yang, Zhuang-Ju Xu, Juan Miao, Ying Bai, Tao Lin

**Affiliations:** School of Life Sciences and Food Engineering, Hebei University of Engineering, Handan 056038, China; sunlingcnu@gmail.com (L.S.); wangyan2022311@163.com (Y.W.); 15175205194@163.com (M.Y.); xu19943514429@126.com (Z.-J.X.); miaojuan220080111@163.com (J.M.)

**Keywords:** blastocyst formation, blastocyst quality, transcription factor, DNA damage, blastocyst hatching

## Abstract

The purpose of this study was to investigate the influence of blastocyst formation timing on the quality of porcine embryos derived from parthenogenetic activation. Newly formed blastocysts at days 6, 7, and 8 of culture [termed formation 6, 7, and 8 blastocysts (F6, F7, and F8 blastocysts)] were obtained, and a series of parameters related to the quality of blastocysts, including apoptosis incidents, DNA replication, pluripotent factors, and blastocyst hatching capacity, were assessed. Delayed blastocyst formation (F7 and/or F8 blastocysts) led to increased levels of ROS, DNA damage, and apoptosis while decreasing the mitochondrial membrane potential, DNA replication, Oct4 levels, and numbers of Sox2-positive cells. F7 blastocysts showed a significantly reduced hatching rate compared to F6 blastocysts; however, F8 blastocysts were unable to develop to the hatching stage. Collectively, our findings suggest a negative correlation between delayed blastocyst formation and blastocyst quality.

## 1. Introduction

In in vitro embryo production experiments, the blastocyst formation rate and/or blastocyst quality are often considered valuable reference indices to evaluate the merits of the in vitro culture system [1,2,3]. However, the different timings of blastocyst formation and collection significantly affect the quality of embryos.

**Figure 1:**
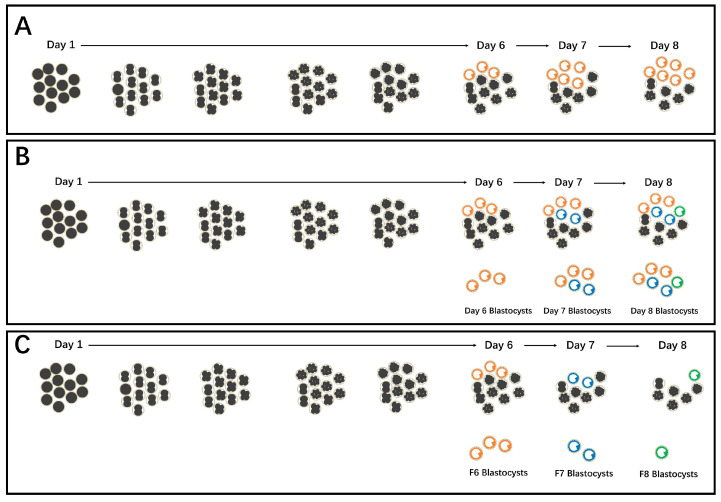
Schematic representation of blastocyst formation dynamics and blastocyst collection methods. (**A**,**B**) Day 6, 7, or 8 blastocysts were obtained using a traditional blastocyst collection method. (**C**) Newly formed blastocysts on day 6 of culture were collected (F6 blastocysts, orange color). The rest of the embryos were left to culture further, and newly formed blastocysts on day 7 of culture were collected (F7 blastocysts, blue color). Similarly, newly formed F8 blastocysts were collected on day 8 of culture (green color). Usually, blastocysts obtained on day 7 or 8 are mixed blastocysts (**B**), e.g., Day 7 blastocysts included F7 (newly formed on day 7 of culture) and old F6 (blastocysts formed on day 6 of culture but were collected on day 7) blastocysts; Day 8 blastocysts included F8 (newly formed on day 8 of culture), old F7 (blastocysts formed on day 7 of culture but were collected on day 8), old F6 (blastocysts formed on day 6 of culture but were collected on day 8) blastocysts.

Conventionally, blastocysts derived from in vitro production are collected after a specific number of days in culture. For instance, porcine blastocysts obtained on day 6, 7, or 8 of culture are termed Day 6, 7, or 8 blastocysts, respectively (Figure 1A). Previous studies have shown that in porcine parthenogenetic activation (PA) [4,5], in vitro fertilization (IVF), and somatic cell nuclear transfer (SCNT) embryos [6], late-collection blastocysts (e.g., Day 8) exhibit decreased embryo quality when compared to early-collection blastocysts (e.g., Day 6), as evidenced by an increase in the apoptosis index. Similarly, in human IVF-derived embryos, early-collection blastocysts are of greater quality [7,8] and result in higher implantation and pregnancy rates [9,10] than late-collection blastocysts. These studies provide very helpful references for improving the quality of embryos derived in vitro.

However, the developmental progression of embryos is not synchronized under in vitro culture conditions. In pigs, for example, some embryos form blastocysts early, on day 6 of culture (termed F6 blastocysts), while others form blastocysts later, on day 7 (F7 blastocysts) or day 8 of culture (F8 blastocysts) (see Figure 1B,C). Blastocysts harvested via the conventional method on day 7 or 8 often comprise a heterogeneous mixture (Figure 1B). For example, Day 7 blastocysts may include both newly formed blastocysts from day 7 (F7 blastocysts) and older blastocysts from day 6 (blastocysts formed on day 6 of culture but harvested on day 7, also known as F6 blastocysts cultured for an additional day post-blastulation). Using these newly formed F6, F7, or F8 blastocysts for experiments may offer greater precision as compared to using conventionally harvested Day 7 or Day 8 blastocysts, which consist of a mixture of new and old blastocysts. Interestingly, despite F6, F7, and F8 all being newly formed blastocysts obtained on days 6, 7, and 8 of culture, respectively, these blastocysts exhibit distinct morphological and quality differences. For example, delayed blastocyst formation (F7 and/or F8) is associated with increased apoptosis and nuclear condensation rates, along with decreased blastocyst diameters and decreased total and ICM cell numbers, as compared to early-formation F6 blastocysts [11]. Although previous research showed that delayed blastocyst formation reduces blastocyst quality, the underlining mechanisms need to be further elucidated.

To gain further insight into the influence and possible mechanisms of blastocyst formation timing on quality in pigs, in this study, we focus on analyzing the impacts of delayed blastocyst formation on the levels of ROS generation, mitochondrial membrane potential, caspase 3 protein, DNA damage, DNA replication, and pluripotent factors (e.g., Oct4, Cdx2, Sox2, and Nanog), as well as the blastocyst hatching capacity. Additionally, the influence of embryonic cleavage kinetics (early or late cleaving) on blastocyst formation dynamics is evaluated.

## 2. Materials and Methods

### 2.1. Chemicals and Animal Ethics Statement

Unless otherwise indicated, chemical and reagents were purchased from Sigma Chemical Co. (St. Louis, MO, USA). Animal experiments were approved by the Animal Care and Use Committee of Hebei University of Engineering (2019-017).

### 2.2. Oocyte Collection and In Vitro Maturation (IVM)

The ovaries of prepubertal gilts were collected from a local slaughterhouse and transported to the laboratory in PBS solution supplemented with 75 μg/mL potassium penicillin G and 50 mg/mL streptomycin sulfate. Porcine follicular fluid was aspirated from antral follicles (3 to 6 mm in diameter) using an 18-gauge needle. Cumulus–oocyte complexes (COCs) with uniform ooplasm and compact cumulus cell mass were washed three to five times and selected for IVM. Approximately 50–60 COCs were transferred to 500 μL of maturation medium under mineral oil at 38.5 °C with saturated humidity in air containing 5% CO_2_ for 22 h. The maturation medium consisted of TCM-199 supplemented with 3.5 mM D-glucose, 0.57 mM L-cysteine, 0.91 mM sodium pyruvate, 50 μg/mL streptomycin, 75 μg/mL penicillin, 10% porcine follicular fluid, 10 ng/mL epidermal growth factor (EGF), 10 IU/mL hCG, and 10 IU/mL PMSG. After 22 h of culture, the medium was replaced with the same medium lacking both hCG and PMSG, and the oocytes were cultured for an additional 22 h.

### 2.3. Embryo Production and Culture

In vitro matured oocytes were subjected to parthenogenetic activation by means of electrical stimulation. Briefly, denuded oocytes were first equilibrated in 0.3 M D-mannitol containing 0.1 mM MgSO_4_, 0.05 mM CaCl_2_, and 0.01% (wt/vol) polyvinyl alcohol (PVA) and then activated via two direct current pulses of 1.1 kV/cm for 30 μs using an Electro Cell Manipulator 2001 (BTX, San Diego, CA, USA). Following this activation, the samples were transferred into PZM-3 containing 4 mg/mL bovine serum albumin (BSA) and 7.5 μg/mL cytochalasin B (CB) for 5 h to inhibit second polar body extrusion. After 5 h of culture with CB, the embryos were washed and transferred to CB-free culture medium (PZM-3 plus 0.4% BSA) and maintained at 38.5 °C in 5% CO_2_. The day of parthenogenetic activation was designated as day 1 of culture.

### 2.4. Porcine Blastocyst Collection and Definition

Porcine F6, F7, and F8 blastocysts were obtained as described in our previous report [11] unless otherwise indicated. Due to the non-synchronized developmental potential of embryos, some embryos form blastocysts early, on day 6 of culture (some references may designate this as day 5, considering the day of activation or insemination as day 0 of culture). Thus, we initially collected these blastocysts and termed them Formation 6 (F6) blastocysts (refer to Figure 1C). Following the collection of F6 blastocysts, the remaining embryos were cultured further to obtain newly formed blastocysts on day 7 of culture, termed Formation 7 (F7) blastocysts (Figure 1C). Similarly, after the collection of F7 blastocysts, the remaining embryos were cultured to obtain newly formed blastocysts on day 8 of culture, termed Formation 8 (F8) blastocysts (Figure 1C). Thus, the F6, F7, and F8 blastocysts represent newly formed blastocysts on days 6, 7, and 8 of culture, respectively.

### 2.5. Reactive Oxygen Species (ROS) Level Measurement

For the analysis of oxidative stress/intracellular ROS levels, H2DCFDA ( 2’,7’-dichlorodihydrofluorescein diacetate; Invitrogen, Eugene, OR, USA) was used to detect ROS as green fluorescence signals. Briefly, porcine blastocysts were incubated at 38.5 °C for 30 min in PBS containing 0.1% PVA (PBS-PVA) and 10 μM H2DCFDA. After the incubation, samples were washed with PBS-PVA, placed into 20 μL PBS-PVA droplets, and examined under an epifluorescence microscope (Olympus, Tokyo, Japan,) with ultraviolet filters (460 nm for ROS, green fluorescence). The same scan settings for each sample were used to normalize the results across the replicates. Fluorescent images were acquired and saved as graphic files in the .TIFF format. The fluorescence intensities were analyzed using ImageJ software (version 1.46r; National Institutes of Health, Bethesda, MD, USA) after background subtraction.

### 2.6. Detection of Mitochondrial Membrane Potential via JC-1 Staining

The mitochondrial membrane potential of blastocysts was investigated using JC-1 dye (T3168; Thermo Fisher Scientific, Eugene, OR, USA). In brief, blastocysts were washed and then exposed to PBS-PVA containing 10 μg/mL JC-1 at 38.5 °C for 30 min. The samples were washed with PBS-PVA to remove surface fluorescence and then examined under an epifluorescence microscope (Olympus, Japan) with the same scan settings for each sample. The mitochondrial membrane potential was analyzed as the ratio of red florescence (J-aggregates, corresponding to activated mitochondria) to green florescence (J-monomers, corresponding to less-activated mitochondria). The red/green fluorescence intensities were quantified, and the background value was subtracted using ImageJ software.

### 2.7. Terminal Deoxynucleotidyl Transferase-Mediated dUTP Nick-End Labelling (TUNEL) Assay

Apoptosis in the blastocysts was investigated by means of TUNEL assay using an in situ Cell Death Detection Kit (TMR Red; Roche, Germany). In brief, blastocysts were fixed in 4% paraformaldehyde for 30 min and then permeabilized with 0.5% Triton X-100 (T9284; Sigma-Aldrich, Dublin, Ireland) for 30 min. After a brief rinse, the samples were reacted with TUNEL for 1 h at 38.5 °C in the dark. Samples were washed in PBS-PVA, and then the nuclei in the blastocysts were labeled with DAPI (Vector Laboratories, Burlingame, CA, USA). Finally, the samples were mounted on glass slides, investigated, and imaged using a Zeiss laser scanning confocal microscope (LSM5 Live, Carl Zeiss, Germany). The rate of apoptosis reflects the percentage of apoptotic cells out of the total number of cells in the blastocysts.

### 2.8. General Immunofluorescence Staining

Porcine blastocysts were fixed in paraformaldehyde (4% in PBS-PVA) for 30 min, permeabilized with 0.5% (*v*/*v*) Triton X 100 for 1 h, blocked with 3% BAS for 1 h, washed in PBG (PBS containing 0.5% BSA and 0.1% gelatin; Sigma) for 20 min, and incubated overnight at 4 °C with primary antibodies. After washing twice for 10 min each time in PBG, the samples were reacted with secondary antibodies for 1 h in the dark. The samples were washed with PBG for 20 min and then mounted using VECTASHIELD mounting medium containing DAPI (H-1200-10; Vector Laboratories, Burlingame, CA, USA) for DNA visualization. The samples were detected and images were captured using a Zeiss laser scanning confocal microscope equipped with a 20× objective lens and running Zeiss LSM Image Browser software. The primary antibodies used were as follows: Anti-CDX2 antibody (1:200, ab76541; Abcom); Oct-3/4 antibody (1:200, sc-5279; Santa Cruz Biotechnology, Oregon, CA, USA); Sox-2 antibody (1:200, sc-365823; Santa Cruz Biotechnology); Nanog antibody (1:200, sc-293121; Santa Cruz Biotechnology); Rad51 antibody (1:200, sc-8349; Santa Cruz Biotechnology); Anti-phospho-Histone H2A.X (Ser139), clone JBW301 (1:200, 05-636; Merck KGaA, Darmstadt, Germany); caspase-3 antibody (1:200, sc-7272; Santa Cruz Biotechnology). The secondary antibodies used were as follows: donkey anti-rabbit FITC (1:200, ab6798; Abcam, Georgia, USA); goat anti-mouse TR (1:200, ab6787; Abcam); goat anti-mouse IgG-FITC (1:200, sc-2010; Santa Cruz Biotechnology); bovine anti-rabbit TR (1:200, sc-2787; Santa Cruz Biotechnology).

### 2.9. EU Staining

EU staining was performed using Click-iT RNA Imaging kits (C10330, Invitrogen) according to the kit instructions. Briefly, blastocysts were cultured in PZM-3 medium supplemented with 1 mM 5-ethynyl uridine (EU) for 1 h. The samples were fixed in 4% paraformaldehyde for 15 min and then permeabilized in 0.5% (*v*/*v*) Triton X-100 for 15 min. After washing with PBS-PVA, samples were reacted with Click-iT reaction cocktail (containing Alexa Fluor 594 azide, C10330; Invitrogen, Glasgow, UK) for 30 min at room temperature, protected from light. The samples were washed with PBS-PVA and then mounted in VECTASHIELD mounting medium containing DAPI for DNA visualization. Images were captured using a Zeiss laser scanning confocal microscope.

### 2.10. EdU Labeling

For EdU labeling, Click-iT EdU Imaging kits (C10337, Invitrogen) were used according to the kit instructions. Briefly, porcine blastocysts were incubated in PZM-3 medium supplemented with 10 μΜ EdU solution for 3 h. The samples were then fixed in 4% paraformaldehyde for 15 min, washed with 3% BSA, and permeabilized with 0.5% Triton X-100 for 20 min. After washing with 3% BSA in PBS, the samples were reacted with Click-iT reaction cocktail containing Alexa Fluor 488 azide as one of the components. After EdU detection, the samples were mounted in VECTASHIELD mounting medium containing DAPI for DNA visualization. Images were captured using a Zeiss laser scanning confocal microscope.

### 2.11. Fluorescence Intensity Analysis

For the fluorescence intensity evaluation, the same instrument settings were adopted throughout to permit an analysis of the data from several replicates. The fluorescence intensities were analyzed using Image J (National Institutes of Health, Bethesda, MD, USA) after background subtraction. For the assessment of ROS, JC-1, caspase-3, and LC3, the average pixel intensity of the blastocysts was calculated using Image J software [12]. A quantitative analysis of H2A.X, RAD51, Oct 4, and EU levels in the nuclei was performed as previously described [13].

### 2.12. Statistical Analyses

Statistical analyses were performed using SPSS 17.0 (SPSS Inc., Chicago, IL, USA). All data were compared via one-way ANOVA followed by Fisher’s protected least significant difference test. Percentage data were subjected to arcsine transformation prior to analysis. At least three replicates were performed for each experiment. The data are expressed as the mean ± se. Any difference with *p* < 0.05 was considered statistically significant.

## 3. Results

### 3.1. The Impact of Delayed Blastocyst Formation on ROS Generation, Mitochondrial Membrane Potential, DNA Damage, and Apoptotic Incidents

**Figure 2:**
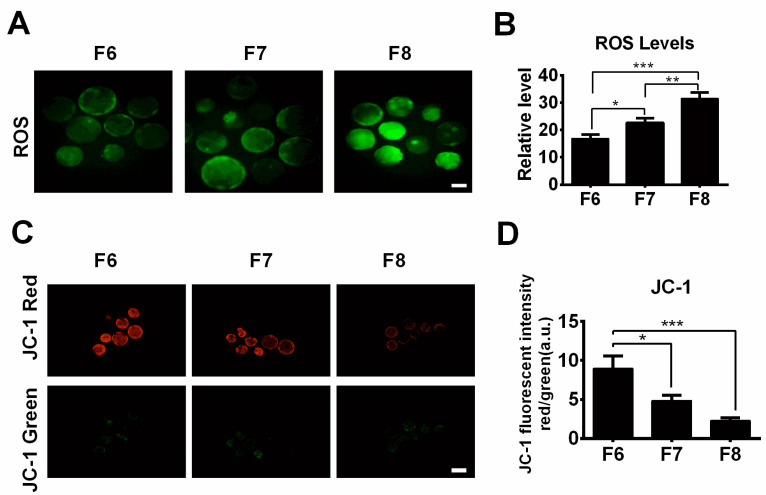
The impact of delayed blastocyst formation on ROS formation and mitochondrial membrane potential. (**A**) ROS staining using H2DCFDA in F6, F7, and F8 blastocysts. (**B**) The relative green fluorescence intensity of ROS was measured in F6 (*n* = 30), F7 (*n* = 30), and F8 (*n* = 28) blastocysts. (**C**) JC-1 staining (J-aggregates, red; J-monomers, green) in F6, F7, and F8 blastocysts. (**D**) The mitochondrial membrane potential was analyzed as the ratio of red to green fluorescence in F6 (*n* = 27), F7 (*n* = 24), and F8 (*n* = 20) blastocysts. * *p* < 0.05, ** *p* < 0.01, *** *p* < 0.001. Scale bars represent 100 μm in (**A**,**C**).

It is well-known that the prolonged exposure of embryos to in vitro culture conditions can lead to a significant increase in ROS production [14]. To investigate whether delayed blastocyst formation leads to enhanced ROS levels, F6, F7, and F8 blastocysts derived from parthenogenetic activation were stained with H2DCFDA, and the levels of ROS generation were analyzed using Image J software. As can be seen in Figure 2A,B, ROS levels exhibited a gradual increase across the blastocyst groups in the order F6 < F7 < F8, as expected. Considering the pivotal role of ROS in the apoptosis process, we further evaluated apoptosis-related incidents in porcine blastocysts by measuring the mitochondrial membrane potential, DNA damage, and apoptosis index markers. Following JC-1 staining, we found that the mitochondrial membrane potential (ratio of red to green fluorescence) was significantly lower in the F7 and F8 blastocysts compared to the F6 group (Figure 2C,D). However, there was no difference in mitochondrial membrane potential between the F7 and F8 groups (Figure 2C,D).

**Figure 3:**
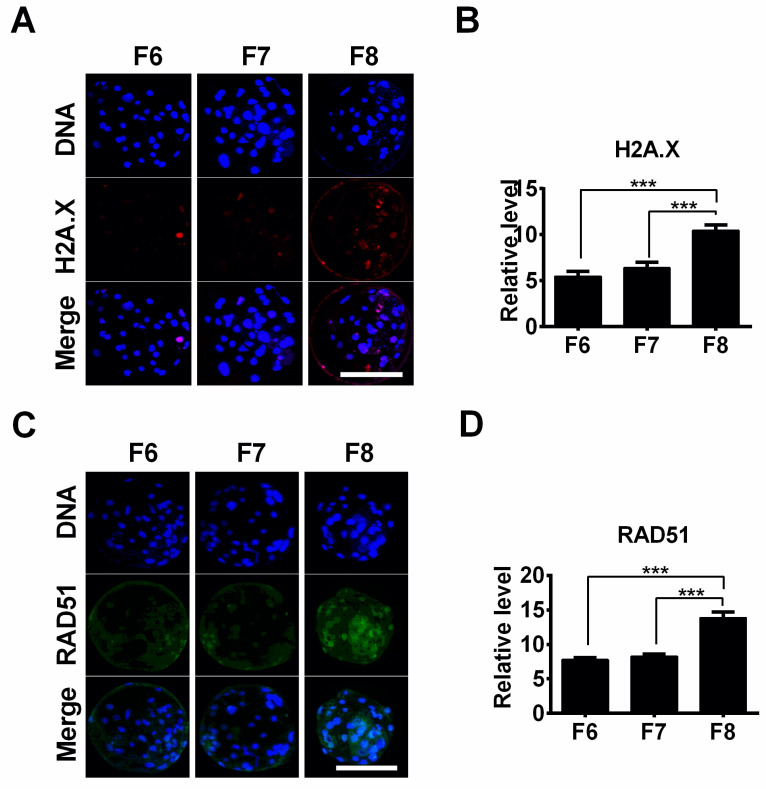
The influence of delayed blastocyst formation on DNA damage. (**A**) DNA damage was evaluated by means of H2A.X immunostaining, and representative images are presented. (**B**) The H2A.X fluorescence intensity was measured in F6 (*n* = 12), F7 (*n* = 12), and F8 (*n* = 12) blastocysts. (**C**) The DNA repair protein RAD51 was tested by means of immunostaining, and representative images are displayed. (**D**) Relative levels of RAD51 were quantified in F6 (*n* = 12), F7 (*n* = 12), and F8 (*n* = 10) blastocysts. *** *p* < 0.001. Scale bars represent 100 μm in (**A**,**C**).

To investigate the influence of delayed blastocyst formation on DNA damage, the levels of the DNA damage response protein H2A.X (Ser139) were determined. The DNA damage levels in the F8 blastocyst group were significantly higher than those in the F6 and F7 groups (Figure 3A,B). However, there were no differences between the F6 and F7 blastocyst groups (Figure 3B). We also evaluated the expression levels of the DNA repair protein RAD51 by means of immunostaining and laser scanning confocal microscopy. We found that the F8 blastocyst group showed a significantly higher expression level of RAD51 than the F6 and F7 blastocyst groups (Figure 3C,D).

**Figure 4:**
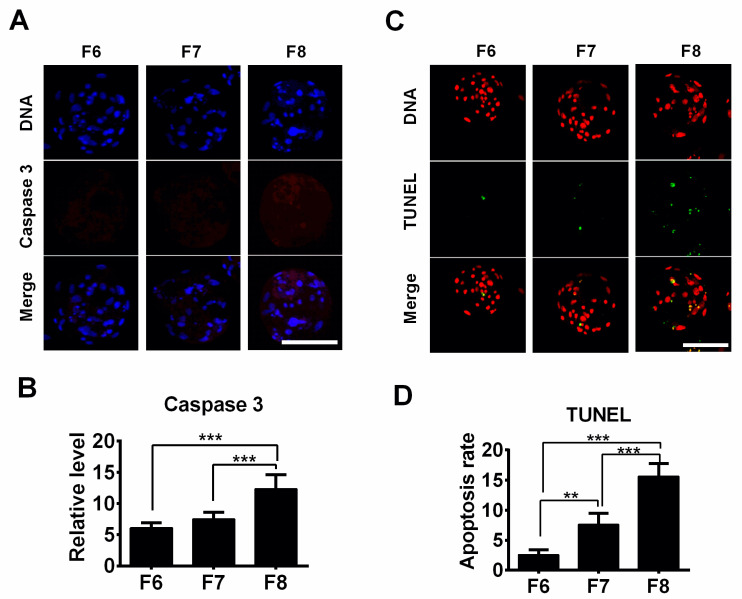
The effect of delayed blastocyst formation on apoptosis in blastocysts. (**A**) Images of caspase 3 immunostaining were obtained via laser scanning confocal microscopy. (**B**) The relative intensity of caspase 3 was quantified in porcine F6 (*n* = 24), F7 (*n* = 24), and F8 *(n* = 24) blastocysts. (**C**) Porcine blastocysts were subjected to TUNEL (green) staining for the detection of apoptosis; DNA was stained with DAPI (red) for better visualization. (**D**) Apoptosis rates were measured as the ratio of the TUNEL-positive cell number to the total cell number (F6, *n* = 25; F7, *n* = 24; F8, *n* = 21). ** *p* < 0.01, *** *p* < 0.001. Scale bars represent 100 μm in (**A**,**C**).

Finally, we investigated apoptotic incidents in the porcine F6, F7, and F8 blastocysts. Although there was no difference in caspase 3 protein levels between the F6 and F7 blastocyst groups, caspase 3 protein levels exhibited a significant increase in the F8 blastocyst group compared to the F6 and F7 blastocyst groups (Figure 4A,B). The apoptosis rates (TUNEL-positive signals) sharply increased across the groups in the order F6 < F7 < F8 (Figure 4C,D). Taken together, these findings suggest that delayed blastocyst formation leads to increased ROS formation and apoptosis-related events.

### 3.2. The Influence of Delayed Blastocyst Formation on Pluripotent Factors

**Figure 5:**
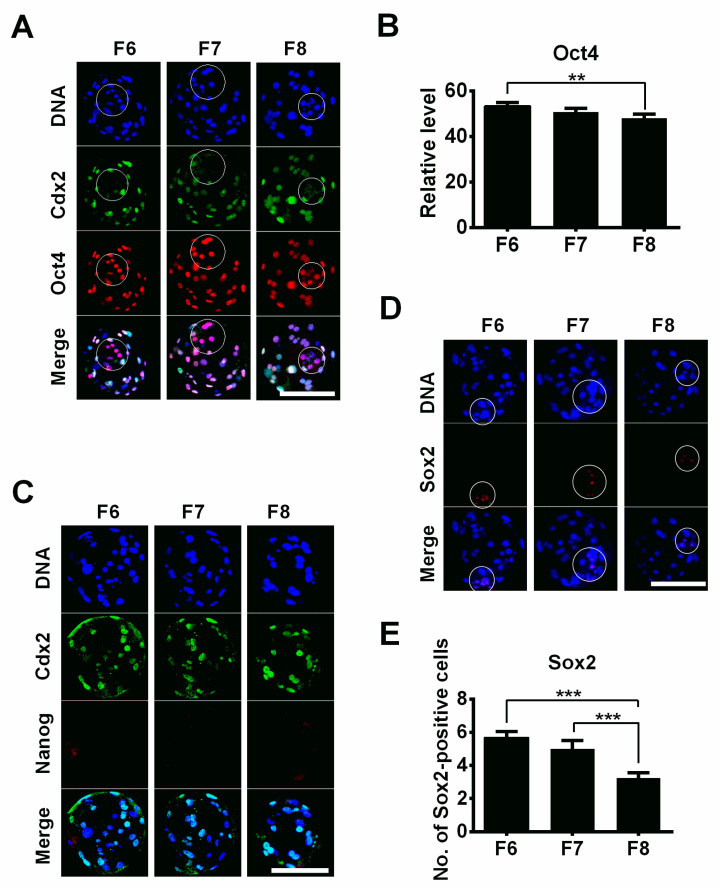
The influence of delayed blastocyst formation on pluripotent factors. (**A**) The expression patterns of Oct4 and Cdx2 were investigated in porcine F6, F7, and F8 blastocysts. (**B**) Quantification of Oct4 levels in porcine blastocysts (F6, *n* = 18; F7, *n* = 18; F8, *n* = 16). (**C**) The expressions of Cdx2 and Nanog were assessed in porcine blastocysts. (**D**) The expression pattern of Sox2 was estimated in blastocysts. (**E**) The numbers of Sox2-positive cells were estimated in blastocysts (F6, *n* = 25; F7, *n* = 24; F8, *n* = 22). ** *p* < 0.01, *** *p* < 0.001. Scale bars represent 100 μm in (**A**,**C**,**D**).

In both early-formation (F6) and late-formation blastocysts (F7 and F8), the Oct4 protein exhibited a co-localized expression pattern in both ICM and TE cells (Figure 5A). However, we observed a decrease in the expression of Cdx2 in several nuclei in the blastocysts (Figure 5A, circle), suggesting that the Cdx2 expression level was starting to decrease in the ICM cells. Although there was no difference in Oct4 expression between the F6 and F7 blastocyst groups, Oct4 levels were significantly lower in the F8 blastocyst group than in the F6 blastocyst group (Figure 5B), indicating that delayed blastocyst formation leads to a decrease in Oct4 expression levels. No Nanog signals were detected in the porcine blastocysts (Figure 5C); however, Sox2 was specifically expressed in the ICM cells (Figure 5D), indicating that Sox2 is an authentic marker of pluripotency in pig blastocysts [15]. The number of Sox2-positive cells (ICM cells) was significantly lower in the F8 blastocyst group than in the F6 and F7 groups (Figure 5E). This result suggests that a delay in blastocyst formation reduces the pluripotency of blastocysts.

### 3.3. The Effect of Delayed Blastocyst Formation on DNA Replication and RNA Transcription

**Figure 6:**
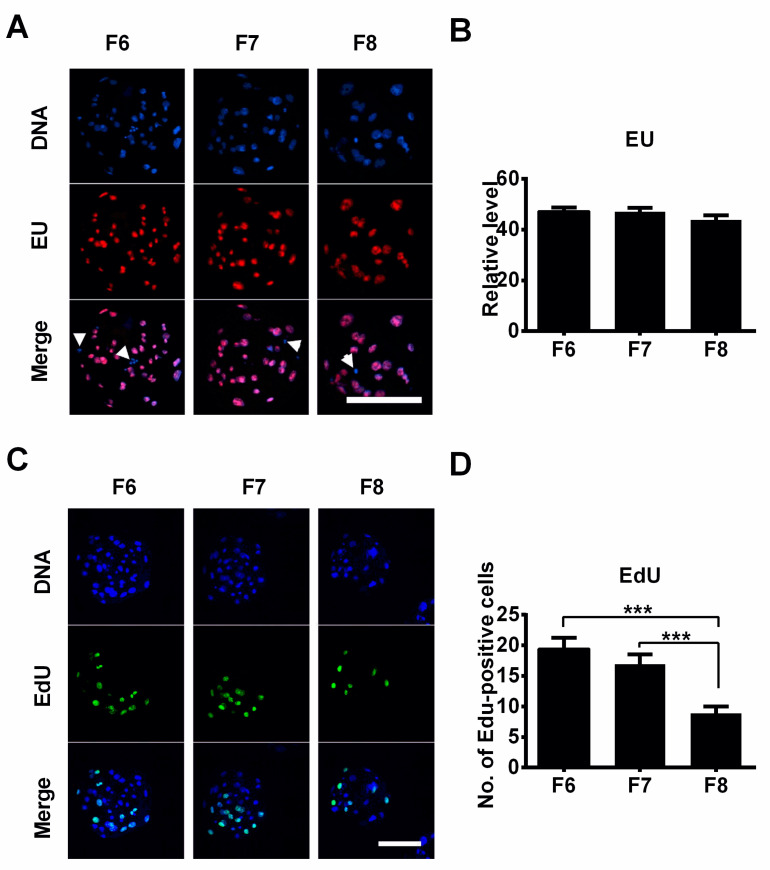
The effect of delayed blastocyst formation on RNA transcription and DNA replication. (**A**) Porcine blastocysts were stained with EU to detect RNA transcription, and representative images were acquired by means of confocal microscopy. (**B**) Transcriptional levels (EU fluorescence intensity levels) were quantified in porcine blastocysts (F6, *n* = 20; F7, *n* = 20; F8, *n* = 16). White arrows indicate M-phase cells. (**C**) Porcine blastocysts were stained with EdU to detect DNA replication, and images were acquired by means of confocal microscopy. (**D**) The numbers of EdU-positive cells were counted in the blastocysts (F6, *n* = 24; F7, *n* = 21; F8, *n* = 17). *** *p* < 0.001. Scale bars represent 100 μm in (**A**,**C**).

To investigate RNA transcription expression in porcine blastocysts, F6, F7, and F8 blastocysts were treated with EU for 1 h. After fixation, permeabilization, and Click-iT detection, the samples were mounted and examined under a laser scanning confocal microscope. EU-positive staining signals were only co-localized in interphase cells and were not observed in M-phase cells (Figure 6A, white arrows), as described in porcine fetal fibroblast cells [16]. There was no difference in nascent RNA synthesis among these three blastocyst groups (Figure 6B), indicating that a delay in blastocyst formation did not influence RNA transcription. Next, we examined the influence of delayed blastocyst formation on DNA replication using 5-ethynyl-2’-deoxyuridine (EdU) staining. Proliferative cells (S phase) were labeled with EdU (green), and all nuclei were stained with DAPI (blue) (Figure 6C). We observed that the numbers of EdU-positive cells were significantly lower in the F8 blastocyst group than in the F6 and F7 groups (Figure 6C,D). The EdU-positive cell numbers in the F6 blastocyst group were higher than those in the F7 group, but this difference was not significant (Figure 6D). Therefore, these results suggest that delayed blastocyst formation leads to a decrease in the DNA replication level but does not influence RNA synthesis.

### 3.4. The Influence of Delayed Blastocyst Formation on Blastocyst Hatching

**Figure 7:**
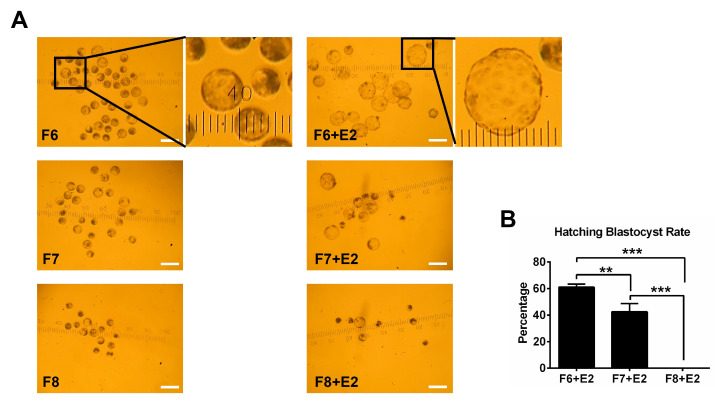
The influence of delayed blastocyst formation on blastocyst hatching. (**A**) Images of blastocyst morphology. (**B**) Hatching rates of blastocysts for the F6 + E2 (*n* = 72), F7 + E2 (*n* = 51), and F8 + E2 (*n* = 30) groups. ** *p* < 0.01, *** *p* < 0.001. The scale bar represents 300 μm in A.

To evaluate the competence of blastocysts from developing to hatching, F6, F7, and F8 blastocysts were transferred to PZM-3 medium supplemented with 10% fetal bovine serum for two extra days of culture (F6 + E2, F7 + E2, and F8 + E2, respectively). We found that the blastocyst hatching rate was dramatically decreased in the F7 + E2 group compared to that in the F6 + E2 group (Figure 7A,B). Importantly, F8 blastocysts only expanded slightly after the two additional days of culture and could not escape from the zona pellucida (Figure 7A,B). These data suggest that a delay in blastocyst formation reduces hatching rates.

### 3.5. The Influence of Embryo Cleavage Kinetics on Blastocyst Formation Dynamics

**Figure 8:**
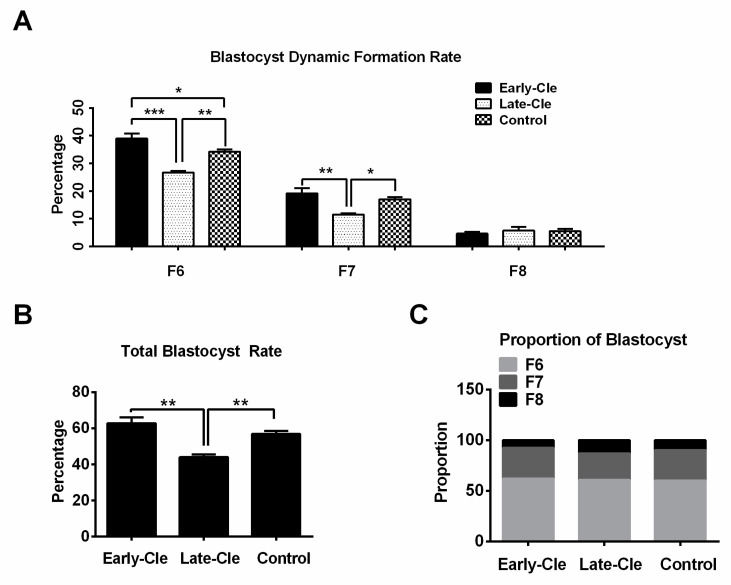
The influence of early and late cleavage on delayed blastocyst formation. (**A**) The dynamic formation rates of blastocysts derived from early-cleavage, late-cleavage, and control groups. (**B**) The total blastocyst formation (F6 + F7 + F8) rates. (**C**) The proportions of F6, F7, and F8 blastocysts in the total blastocyst group (F6 + F7 + F8). In total, 151, 149, and 146 cleaved embryos derived from early-cleavage embryos, late-cleavage embryos, and a control group were used for evaluation. Early-Cle and Late-Cle indicate the early-cleavage and late-cleavage embryo groups. * *p* < 0.05, ** *p* < 0.01, *** *p* < 0.001.

Studies in humans [17], mice [18], bovines [19], and pigs [20] have suggested that early-cleavage embryos provide higher blastocyst formation rates and better blastocyst quality than late-cleavage embryos. This led us to postulate that embryo cleavage kinetics might influence blastocyst formation dynamics. To this end, porcine in vitro maturated oocytes were activated by means of electrical stimulation, and then early-cleavage (before 24 h), late-cleavage (24–48 h), and normal control (a combined group where early- and late-cleavage embryos were not identified) embryos were obtained and cultured in PZM-3 plus 4% BSA. The F6 and F7 blastocyst rates were significantly lower in the late-cleavage embryo group compared to the early-cleavage and control groups (Figure 8A). However, no difference was observed in the F8 blastocyst rates among the early-cleavage, late-cleavage, and control embryo groups (Figure 8A). Although the early-cleavage embryos exhibited a higher yield of F6 blastocysts compared to the control group (*p* < 0.05) (Figure 8A), no difference was observed in the total blastocyst (F6 + F7 + F8) rate between the early-cleavage embryos and the control group (Figure 8B). However, the total blastocyst rate was significantly lower in the late-cleavage group compared to both the early-cleavage and control groups (Figure 8B). Importantly, we noticed that although late-cleavage embryos exhibited significantly reduced rates of F6 and F7 blastocysts (Figure 8A) and total blastocyst rates (Figure 8B) when compared to the early-cleavage and control groups, the proportions of F6, F7, and F8 blastocysts in the total blastocyst group (F6 + F7 + F8) remained unaffected across the early-cleavage, late-cleavage, and control groups (Figure 8C).

## 4. Discussion

To elucidate the potential mechanisms underlying the influence of delayed blastocyst formation on blastocyst quality, newly formed F6, F7, and F8 blastocysts were obtained, and a series of parameters related to embryo quality were evaluated. Our results indicate that a delay in blastocyst formation led to increased levels of ROS, DNA damage, and caspase 3 protein. Additionally, there was a decrease in the mitochondrial membrane potential, Oct 4 expression level, and hatching capacity of the blastocysts.

Oxidative stress plays an important role in cellular apoptosis [21,22]. ROS are some of the main products of oxidative reactions. In our study, the data revealed that intracellular ROS levels increased in a time-dependent manner with delayed blastocyst formation. In the current study, a temperature of 38.5 °C with saturated humidity in air containing 5% CO_2_ was used in the culture. This condition could be a key factor in ROS production and represents a limitation of the study. Therefore, to obtain more accurate information, it might be better to use 5% O_2_ instead. The overproduction of ROS can induce mitochondrial permeability transition [23], thereby decreasing the mitochondrial membrane potential. This reduction in the mitochondrial membrane potential can lead to disruption of the mitochondrial membrane, resulting in the release of cytochrome c from mitochondria. This event can trigger a caspase cascade, ultimately inducing cell apoptosis [24,25]. Our JC-1 staining assay suggested that the mitochondrial membrane potential was significantly reduced in the F7 and F8 blastocyst groups compared to the F6 blastocyst group, indicating that the mitochondrial membrane may have been ruptured in these delayed blastocysts. Mitochondria are the main source of ROS in cells [26]. Thus, as soon as mitochondria encounter damage, net ROS formation is increased as a result of an imbalance between the formation and elimination of ROS inside the cells [27]. This might explain why higher ROS levels were observed in the F7 and F8 blastocysts. In the current study, our data showed that delayed blastocyst formation was associated with increased oxidative stress, subsequently resulting in cell apoptosis. This was confirmed by the accumulation of intracellular ROS levels, up-regulated expression of caspase 3 protein, increased numbers of TUNEL-positive cells, and decreased mitochondrial membrane potential. In addition, a major consequence of DNA damage is cellular apoptosis or death [28,29]. In our study, immunostaining using H2A.X and RAD51 antibodies revealed that delayed blastocyst formation was associated with significantly increased DNA damage levels, consistent with our previously observed findings regarding apoptosis.

In mammalian preimplantation embryonic development, the transcription factors Sox2, Oct4, and Nanog play important roles as fundamental regulators maintaining the self-renewal of pluripotent ICM cells [30]. In mice and cattle, Cdx2 and Oct4 expression can be detected specifically in TE and ICM cells [13,31,32], respectively. In contrast, in pigs, Cdx2 is specifically expressed in TE cells and downregulated in ICM cells [33]. However, Oct4 expression is maintained at high levels in TE and ICM cells of porcine blastocysts [15,34]. Our current results confirmed that the Oct4 protein was expressed in both ICM and TE cells, and Cdx2 was specifically expressed in TE cells of porcine blastocysts. The expression levels of the Oct4 protein showed a gradual downregulation from F6 blastocysts to F8 blastocysts, suggesting that a delay in blastocyst formation reduced the quality of blastocysts by decreasing the Oct4 expression level. In addition, we failed to detect Nanog protein expression in any blastocyst group, supporting the evidence from other studies that Nanog expression is undetectable in porcine blastocysts [15,33]. Unlike Oct4 or Nanog, the Sox2 protein is restricted to the ICM cells of porcine blastocysts (including F6, F7, and F8 blastocysts), suggesting that Sox2 serves as a faithful marker of pluripotency in porcine blastocysts [15]. The number of Sox2-positive cells was significantly lower in the F8 blastocysts compared to the F7 and F6 groups, indicating that a delay in blastocyst formation reduced the number of ICM cells.

The total cell number of a blastocyst serves as a key indicator of its quality [35]. In the current study, to evaluate the capacity for cell proliferation in blastocysts, we utilized EdU staining. Our results revealed that the number of EdU-positive cells was significantly lower in the F8 blastocysts compared to the F6 and F7 blastocysts, suggesting that a delay in blastocyst formation can lead to a reduction in cell proliferation. This may explain why the F8 blastocysts contained fewer total cells than the F7 and F6 blastocysts. However, the precise mechanism by which delayed blastocyst formation influence cell proliferation in blastocysts requires further investigation.

Our next objective was to enhance the ability of embryos to develop into early-formation (F6) blastocysts, as these early-formation blastocysts are of higher quality compared to late-formation blastocysts. Accumulating evidence has demonstrated that the timing of the initial cleavage event is correlated with the potential of embryos to develop into a blastocyst. Early-cleavage embryos are more likely to develop into blastocysts than late-cleavage embryos [18,20,36,37,38]. In the present study, our data suggest that embryos’ cleavage kinetics are related to their subsequent blastocyst formation dynamics. Early-cleavage embryos exhibited significantly improved total blastocyst formation rates and enhanced F6 and F7 blastocyst rates when compared to late-cleavage embryos. Thus, our results support the evidence that early-cleavage embryos provide higher-quality blastocysts and a higher rate of blastocyst formation compared to late-cleavage embryos. Furthermore, we analyzed the proportions of F6, F7, and F8 blastocysts in the total blastocyst group (F6 + F7 + F8). Interestingly, embryo cleavage kinetics did not influence the proportions of F6, F7, and F8 blastocysts. The mechanisms and causes of delayed blastocyst formation need further study.

In conclusion, our study showed a negative correlation between delayed blastocyst formation and blastocyst quality in pigs. A delay in blastocyst formation reduces blastocyst quality by increasing the ROS levels, DNA damage, and apoptosis index while decreasing the mitochondrial membrane potential, DNA replication, and embryonic pluripotency, as well as reducing the developmental capacity to reach the hatching stage. Early-cleavage embryos exhibited significantly increased rates of both F6 and F7 blastocysts, as well as increased total blastocyst rates (F6 + F7 + F8), when compared to late-cleavage embryos. However, the kinetics of embryo cleavage did not affect the proportions of F6, F7, and F8 blastocysts within the total blastocyst group.

## Data Availability

All the data are presented in the manuscript.
